# Heterozygous Yeast Deletion Collection Screens Reveal Essential Targets of Hsp90

**DOI:** 10.1371/journal.pone.0028211

**Published:** 2011-11-30

**Authors:** Eric A. Franzosa, Véronique Albanèse, Judith Frydman, Yu Xia, Amie J. McClellan

**Affiliations:** 1 Bioinformatics Program, Boston University, Boston, Massachusetts, United States of America; 2 Department of Chemistry, Boston University, Boston, Massachusetts, United States of America; 3 Department of Biology, Stanford University, Stanford, California, United States of America; 4 Division of Natural Sciences and Mathematics, Bennington College, Bennington, Vermont, United States of America; University of Edinburgh, United Kingdom

## Abstract

Hsp90 is an essential eukaryotic chaperone with a role in folding specific “client” proteins such as kinases and hormone receptors. Previously performed homozygous diploid yeast deletion collection screens uncovered broad requirements for Hsp90 in cellular transport and cell cycle progression. These screens also revealed that the requisite cellular functions of Hsp90 change with growth temperature. We present here for the first time the results of heterozygous deletion collection screens conducted at the hypothermic stress temperature of 15°C. Extensive bioinformatic analyses were performed on the resulting data in combination with data from homozygous and heterozygous screens previously conducted at normal (30°C) and hyperthermic stress (37°C) growth temperatures. Our resulting meta-analysis uncovered extensive connections between Hsp90 and (1) general transcription, (2) ribosome biogenesis and (3) GTP binding proteins. Predictions from bioinformatic analyses were tested experimentally, supporting a role for Hsp90 in ribosome stability. Importantly, the integrated analysis of the 15°C heterozygous deletion pool screen with previously conducted 30°C and 37°C screens allows for essential genetic targets of Hsp90 to emerge. Altogether, these novel contributions enable a more complete picture of essential Hsp90 functions.

## Introduction

Hsp90 is an essential and abundant eukaryotic chaperone with an established role in the folding of “client” proteins involved in cellular signaling pathways, primarily kinases and steroid hormone receptors. The specific requirement for Hsp90 in the activation and conformational maintenance of oncogenic kinases critical for cellular transformation [1], [2], [3], as well as its recently discovered role in mediating viral protein folding [4], make Hsp90 an attractive target for therapeutic intervention in cancer and viral pathogenesis. Drug-based inhibition of Hsp90 results in the proteasomal degradation of its defunct folding substrates, thus removing them from the cellular milieu [5], [6], [7], [8]. Hsp90 has also been shown to participate in the cytosolic quality control of proteins that are not its folding substrates [9]. These connections between Hsp90 and quality control, including its ability *in vitro* to prevent protein aggregation [10], as well as its affiliation with Aß, tau, and alpha-synuclein aggregates [11], [12], [13], [14], also suggest that Hsp90 function may be a critical determinant in the onset or severity of neurodegenerative diseases linked to protein misfolding [15].

Hsp90 functions as an ATP-dependent dimer [16], [17]; each protomer of Hsp90 consists of an N-terminal ATP binding domain, a short charged linker connecting to the Middle domain, and a C-terminal dimerization domain. Structurally and mechanistically, Hsp90 is a member of the GHKL (DNA **G**yrase, **H**istidine **K**inase, Mut **L**) family of dimeric ATPases. As recent structural and biochemical studies have demonstrated, the transient ATP-dependent dimerization of Hsp90 N-termini appears to be conserved in the non-essential bacterial homologue HtpG [18], yeast Hsp90 [19], [20], and human Hsp90ß [21], [22], suggesting a conserved ATPase cycling mechanism involving dramatic conformational rearrangements.

The ATPase cycle, substrate interactions, and conformational changes of Hsp90 can all be modulated by the interaction of Hsp90 with various cochaperones. These cochaperones interact with Hsp90 and stimulate ATPase [23], inhibit ATPase [24], [25], [26], or otherwise stabilize certain conformations of Hsp90 and thus facilitate the binding or release of Hsp90 clients. Particular cochaperones influence the specific clientele of Hsp90; for example, kinase recruitment is dependent upon Cdc37/p50 while steroid hormone receptors rely upon Sti1/Hop for transfer to Hsp90 from early folding intermediates on Hsp40/Hsp70 [27], [28]. While some cochaperone interactions with Hsp90 are well characterized, it would be perilous to assume our knowledge of how cochaperones interact with Hsp90 is near completion. For example, while it is well established that the C-terminal MEEVD residues of Hsp90 facilitate interaction with TPR-containing cochaperones, the Hsp90 cochaperone Sgt1p, which contains a TPR motif, does not bind Hsp90 via its TPR domain [29]. Other cochaperones interact with Hsp90 via multiple surface patches (i.e. Sba1/p23; [20]), which makes predicting all possible Hsp90 cochaperones from sequence alone untenable.

Despite recent advances in obtaining full-length structures of Hsp90 and its paralogs [18], [20], [30], some including, and perhaps made possible by, co-crystals of Hsp90 conformation-stabilizing cochaperones [20], [31], general guidelines defining how Hsp90 interacts with its substrates are lacking. While substrate recognition determinants are well-defined for chaperones such as Hsp70 and Hsp60, which bind to linear exposed sequences of hydrophobic amino acids, or to hydrophobic patches in unstructured proteins [32], [33], [34], [35], Hsp90 is thought to interact with its substrates after they have adopted a more structured or quasi-native state [31], [36]. This means that Hsp90-client interaction may be dictated by sequences and structures in disparate regions of the folding substrate and therefore depend on the conformation of the client, as well as on the conformation of Hsp90 when it encounters the client. As an example, one lobe of the kinase client cdk4 straddles the Middle domain of one Hsp90 protomer [31], in accordance with a mutagenesis study showing the importance of the Middle domain in client binding [37] and structural studies of HtpG [18], but HtpG structures additionally suggest that all three Hsp90 domains expose interdomain hydrophobic surfaces accessible to clients when Hsp90 is in the “open”, non N-terminally dimerized, conformation [18]. Altogether, it is clear that the more information we can obtain and analyze with regard to Hsp90 interacting proteins, be they cochaperones or substrates, the more we can learn about how Hsp90 functions, what its essential cellular role(s) are, and how best to target Hsp90 or specific “clients” in attempts to ameliorate disease states.

The now evident conformational flexibility of Hsp90, the transient nature of chaperone-substrate interactions, and the lack of defined Hsp90-client binding sequences and locations have collectively made the identification of Hsp90 cochaperones and substrates somewhat challenging. Nevertheless, recent large-scale studies have drastically increased the known cellular scope of Hsp90 interactors and functions [38], [39], [40]. Chemical-genetic screens of the homozygous diploid yeast deletion collection utilizing a specific Hsp90 inhibiting drug effectively deal with the issues of transient Hsp90 interactions and Hsp90 conformational flexibility as they are conducted over an extended period of ten generations of growth, providing an ample time frame to capture perhaps otherwise overlooked interactions [40]. These screens uncovered significant requirements for Hsp90 in various aspects of cellular transport and secretion, and in cell cycle progression. Interestingly, these screens also revealed that the requisite cellular functions of Hsp90 change according to whether yeast are subjected to normal or stress-inducing growth temperatures.

To extend the findings of the homozygous deletion pool screens, chemical-genetic screens of the heterozygous diploid deletion collection were also performed. Heterozygous deletion pool screens differ from homozygous deletion pool screens in two important ways. First, heterozygous deletion pool screens can readily identify essential cellular substrates of Hsp90. Second, screening for deletion strains sensitive to the loss of Hsp90 function may involve a different mechanism in the half-dosage background of the heterozygous deletion strains, which can be elucidated by comparing the homozygous and heterozygous deletion pool data sets. In a previous study, we inhibited Hsp90 function at the normal growth temperature of 30°C and under conditions of hyperthermic stress [40].

Here, we report the findings of conducting the heterozygous deletion pool screen at the hypothermic stress temperature of 15°C. Further, we perform extensive analyses of the heterozygous deletion screens performed at 15°C, 30°C, and 37°C. Finally, we conduct a meta-analysis of all homozygous and heterozygous deletion pool screens, along with combined analysis of all curated Hsp90 interactors, permitting us to build a complete picture of Hsp90 function in yeast. These analyses critically examine unnamed ORFs identified in these screens, identify novel essential cellular targets that may underlie the necessity of Hsp90 for viability, identify critical hubs of Hsp90 action in cellular processes, and allow genome-wide predictions of *bona fide* Hsp90 substrates and chaperones, all of which further contribute to our knowledge of what governs Hsp90-substrate and Hsp90-cochaperone interactions.

## Results

In this paper, an Hsp90 “interaction” is defined as a significant growth defect in the chemical genetic screen, indicative of a broad genetic interaction. Terminology such as “interactor” and “target” are all defined based on these genetic interactions and, unless directly stated, not meant to imply a specific type of interaction (i.e. genetic versus physical).

### Broad analyses of heterozygous deletion pool data sets

**Figure 1:**
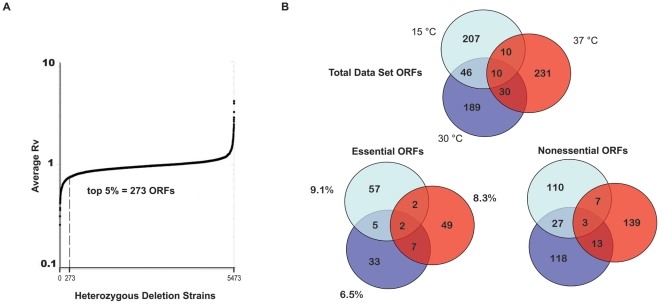
Overview of 15°C Data Set Generation and General Composition of Heterozygous Deletion Pool Data Sets. **A.** Pooled heterozygous diploid deletion strains comprising most yeast ORFs were grown for 10 generations at 15°C in the absence or presence of Macbecin II. Each deletion strain is plotted versus its average Ratio Value (Rv) from three independent experiments. The top 5% most sensitive deletion strains were selected as the data set for further study (average Rv≤0.726; 273 ORFs). **B.** The top Venn Diagram shows overlap amongst the three temperature heterozygous data set deletion strains (includes nonessential and essential ORFs). The bottom left Venn Diagram shows overlap amongst essential heterozygous deletion strains. The bottom right Venn Diagram shows overlap amongst only nonessential heterozygous data set deletion strains that were tested but not sensitive to Hsp90 inhibition in the context of homozygous deletion.

Pooled heterozygous yeast deletion strains were subjected to ten generations of growth in the absence or presence of macbecin II, as previously described [40], except at a hypothermic stress-inducing temperature of 15°C. In our previous study, we showed that the top 5% homozygous deletion strains most sensitive to Hsp90 inhibition were significantly enriched for established Hsp90 interactors, thus validating their potential to reveal novel Hsp90 substrates and cochaperones [40]. Since the effects of Hsp90 inhibition in heterozygous deletion strains may be less pronounced due to half gene dosage effects, we first examined whether the top 5% most sensitive ORFs in the heterozygous deletion pool experiments were statistically comparable to the homozygous top 5% data sets. Interestingly, the heterozygous data sets, whether using a cutoff of the top 5%, 7.5%, 10%, or 15% most growth-inhibited strains, never approached the statistically significant enrichment of established Hsp90 interactors observed for the homozygous deletion pool experiments. In fact, only the heterozygous 37°C experiments showed significant enrichment at any cutoff used (p-values of 1×10^−3^ and 6×10^−3^ for cutoffs of top 5% and 7.5%, respectively; Table S1). Thus, in order to enable the most comparable analysis to our previously defined homozygous and heterozygous 30°C and 37°C data sets, we selected the top 5% most sensitive heterozygous deletion strains, comprising 273 ORFs as the 15°C data set for further study (Figure 1A; Table S2; total ranked data for the 15°C screen is found in Table S3).

We first broadly compared the overall ORF composition of the combined heterozygous data sets. While there was some overlap amongst the three data sets, the majority of hypersensitive strains were unique to each temperature (Figure 1B, top Venn diagram). Notably, there was statistically significant overlap between the data sets closer in temperature (15°C/30°C and 30°C/37°C; Fisher's exact test p<0.001), but not between the 15°C and 37°C data sets (p = 0.39); a small subset of ORFs was found in common to all three data sets (Table 1).

**Table 1:** ORFs Occurring in all Three Heterozygous Data Sets (15°C, 30°C, and 37°C).

ORF/Gene Name	Essential	Nonessential	Published Hsp90 Interactions in BioGRID
YBL065W/Dubious ORF		√	0 (no published interactions with any gene/protein)
YDR328C/SKP1	√		3
YDR335W/MSN5		√	1
YGR255C/COQ6			1
YML085C/TUB1	√		1
YNL037C/IDH1		√	1
YOL087C/DUF1		√	1
YOR153W/PDR5		√	1
YOR333C/Dubious ORF		√	0 (no published interactions with any gene/protein)
YPR021C/AGC1	√		1

One important feature of performing these experiments with the heterozygous deletion collection is the ability to examine what essential proteins may require Hsp90 function. Overall, our combined heterozygous data sets contain a total of 155 essential ORFs, of which only 19 have been previously established as Hsp90 interactors (Table S4). Thus, these chemical-genetic screens have uncovered many novel Hsp90 targets encoded by essential genes.

When comparing the overlap amongst only essential heterozygous data set ORFs, again there were more unique strains for each temperature than strains common to multiple experimental conditions. Also, we again observe statistically significant overlap between the data sets closer in temperature (15°C/30°C and 30°C/37°C; p<0.001), but not between the 15°C and 37°C data sets (p = 0.08). Intriguingly, the percentage of essential ORFs was higher for the 15°C (9.1%) and 37°C (8.3%) data sets than for the 30°C data set (6.5%); one possibility suggested by these data is that Hsp90 is more required for essential viability-maintaining functions under suboptimal growth conditions than at the normal growth temperature of 30°C (Figure 1B, lower left Venn diagram).

Another interesting class of heterozygous data set ORFs is comprised of nonessential ORFs that were tested, but not hypersensitive to, Hsp90 inhibition in the context of the homozygous deletion pool screens [40]. We hypothesize that these particular candidates have an increased likelihood of being direct Hsp90 interactors as, in the face of diminished Hsp90 function, their complete absence from the genome resulted in a less severe growth defect than their presence at half gene dosage. As shown in Figure 1B (lower right Venn diagram), numerous heterozygous data set ORFs fulfill this condition; the largest number of condition-specific candidates are affiliated with Hsp90 inhibition at 37°C. Observed overlap between nonessential 15°C/30°C and 30°C/37°C data sets is statistically significant (p<0.001), with weak significance for 15°C/37°C overlap (p = 0.02). Altogether, the analyses of data set overlap consistently show that data sets closer in temperature share more targets and that this is statistically significant. Further, this did not change when analyzing total data set ORFs versus partitioning candidates as essential or nonessential ORFs.

### Analysis of unnamed ORFs in heterozygous data sets

As unnamed ORFs are generally uncharacterized or dubious, there is a lack sufficient information to permit analyses based on, for example, common Gene Ontology function or process classifications. Instead, unnamed data set ORFs were screened for commonalities determined by predicted cellular localization, overall hydrophobicity, predicted degree of disorder, number and type of known interactors, and predicted secondary structure domains. Surprisingly, these analyses yielded no significant information to aid in further classifying these largely uncharacterized ORFs, suggesting that although their half-dosage deletion results in hypersensitivity to Hsp90 inhibition, there is no obvious unifying feature, or features, that elucidates the reason(s) why.

Overall, the unnamed ORFs comprise 17 verified ORFs, 86 uncharacterized ORFs, 80 dubious ORFs, and two merged ORFs. Looking at these ORFs individually reveals that, in many cases, there are testable connections that may underlie their occurrence in these data sets. For example, eight of the unnamed ORFs have some connection to ribosomes due to (i) predicted physical interaction (five verified ORFs), (ii) a predicted role in ribosome biogenesis (one uncharacterized ORF), or (iii) partially overlapping with known ribosomal genes (two dubious ORFs). There is also one unnamed ORF that contains TPR domains and thus has the potential to be an Hsp90 cochaperone. In addition, there are 15 dubious ORFs that partially overlap with named ORFs occurring in the screen data sets, thus giving credence to their identification in these screens despite the lack of evidence that they themselves encode functional proteins. For example, some of these dubious ORFs overlap with *SKP1*, *SPT4*, or *SIN4*, which are involved, respectively, in kinetochore assembly, kinetochore function and RNA Pol I and III transcriptional regulation, and RNA Pol II Mediator activity. Other dubious ORFs overlap with players in ribosome biogenesis and translation, including *RPL10*, *NOP58*, *SUI3*, and *RPL43A*. Altogether, there is sufficient evidence to indicate that dubious ORFs should not be discounted as unimportant to this analysis simply because they themselves are unlikely to encode proteins.

### Insights from integration of multiple experiments

Combining the information acquired from our five experimental screens (homozygous 30°C and 37°C; heterozygous 15°C, 30°C, and 37°C) provides stronger support for novel Hsp90 interactions. The logic here is straightforward: if an ORF appears in multiple sensitive data sets, then our confidence that it is a true Hsp90 interactor grows. One way to quantify this measure of confidence is simply to count the number of screens in which an ORF appears in the sensitive data set. Consistent with our intuition, we find that ORFs appearing in one sensitive data set are 46% more enriched for known Hsp90 interactors than expected by chance (Fisher's exact test, *p*<10^−7^), while ORFs appearing in two sensitive data sets are 115% more enriched than expected by chance (*p*<10^−7^). The number of ORFs appearing in more than two sensitive data sets is too small to provide significant enrichment statistics.

One shortcoming of the counting approach is that each experiment is allotted equal weight. This is not ideal, as some experiments may be inherently more or less successful at identifying known Hsp90 interactors than others. In addition, the counting approach does not differentiate between non-sensitive ORFs and untested ORFs. A more rigorous approach involves calculating likelihood ratios (LRs) for Hsp90 interaction based on the three possible results for each ORF in a given experiment: (1) the ORF was tested and sensitive, (2) the ORF was tested and non-sensitive, and (3) the ORF was not tested. This allows us to effectively normalize our data sets by accounting for the fact that essential ORFs were not tested in the context of the homozygous deletion pool screens. A LR is constructed by comparing all ORFs with a given result in a given experiment to gold standards of Hsp90 interaction and non-interaction:
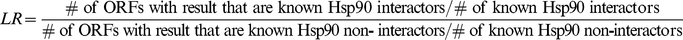
The LR indicates the factor by which the odds of being a known Hsp90 interactor change when its associated experimental result is observed for an ORF. A sensitive result is ideally associated with an increase in the odds of Hsp90 interaction (LR>1), and a tested but non-sensitive result is ideally associated with a decrease in the odds of Hsp90 interaction (0<LR<1). For this analysis, we defined ORFs with two or more reported Hsp90 interactions in BioGRID as gold standards of Hsp90 interaction and ORFs with no reported Hsp90 interactions as gold standards of Hsp90 non-interaction. The LR values for the three possible results in each of the five experiments are reported in Table 2. For example, the LR for sensitivity in the heterozygous 30°C screen is 3.30, meaning that an ORF's odds of being a known Hsp90 interactor more than triple when it is observed as sensitive in this experiment.

**Table 2:** Likelihood Ratios* (LRs) of Hsp90 Interaction For All Five Screen Conditions.

	Hom 30°C	Hom 37°C	Het 15°C	Het 30°C	Het 37°C
**LR (1)** Odds of Hsp90 interaction change by this factor if ORF tested and sensitive	3.72	2.60	1.05	3.30	3.57
**LR (2)** Odds of Hsp90 interaction change by this factor if ORF tested and non-sensitive	0.73	0.79	1.08	0.93	0.94
**LR (3)** Odds of Hsp90 interaction change by this factor if ORF was not tested	1.21	1.25	0.57	0.72	0.60

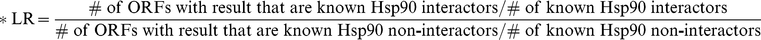

Applying the Naïve Bayes assumption that individual results are conditionally independent, we take the product of the appropriate LR values for each ORF to find the total change in the odds of Hsp90 interaction inferred from the five experimental screens. Relative to a random ORF, the ten ORFs with the most support are all at least 30 times more likely to be known Hsp90 interactors than not. While three of these ORFs have indeed been reported previously as Hsp90 interactors (YIL170W/*HXT12*, YMR070W/*MOT3*, YOR027W/*STI*1), the remaining seven have not (YDR415C, YGL013C/*PDR1*, YDR335W/*MSN5*, YOL050C, YNL235C, YLR330W/*CHS5*, YOR333C). Statistically, it is highly likely that these seven ORFs participate in interactions with Hsp90, even though they have never been previously reported to do so; such ORFs would make excellent candidates for targeted small-scale investigations. We have therefore demonstrated that statistical integration of multiple large-scale experimental screens both increases our confidence in their results and aids in the design of future experiments.

### Predicting Hsp90 target assignments from data set ORF rankings

The underlying methodology used in our chemical-genetic screens of homozygous and heterozygous yeast deletion collections generates highly reproducible and quantifiable results [41]. Thus, this meta-analysis of all five deletion collection screen data sets provides the opportunity to explore whether predictive tools for assigning sensitive ORFs as putative Hsp90 cochaperones or client proteins can be established.

One approach toward this end was to analyze the temperature (15°C, 30°C, 37°C) and condition (homozygous or heterozygous deletion) dependent rankings of high-confidence Hsp90 interactors (Table 3) and attempt to identify specific patterns. The entire ranked data sets can then be scanned for ORFs fitting certain patterns of sensitivity, which would classify targets as specific types of Hsp90 interactors, or as potentially interacting with or in parallel to specific known cochaperones or folding clients. For example, yeast lacking the well-established cochaperone Sti1p [42] were extremely sensitive to Hsp90-inhibition in both homozygous and heterozygous deletion contexts, except for in the heterozygous background at 37°C (Table 3). A similar or unifying pattern for other genes encoding TPR-containing cochaperones (*CNS1, CPR7, CPR6, SGT1, PPT1, TAH1*) was not observed. In fact, there was a wide range of variability, from no significant growth inhibition under any tested condition (*CPR7*), to being more affected in the context of homozygous deletion (*TAH1*), inhibited specifically only at 30°C in the homozygous deletion background (*CPR6*), or inhibited specifically only at 37°C in the heterozygous condition (*PPT1*). The only two similar patterns observed for TPR-containing proteins are for the essential genes *CNS1*, Hsp90 cochaperone and multicopy suppressor of Hsp90 deficiency [43], [44], [45], and *SGT1*, which encodes a cochaperone important for kinetochore assembly [46], [47]; yeast heterozygous for *CNS1* or *SGT1* were specifically sensitive to Hsp90 inhibition only at 30°C.

**Table 3:** Temperature-Dependent Rankings of Well-Established Hsp90 Interactors.

ORF/Gene name	Essential (Y/N)	Type of Interactor	BioGRID Hits	Physical/Genetic	Hom 30°C	Hom 37°C	Het 15°C	Het 30°C	Het 37°C
YOR027W/STI1	N	Cochaperone	25	P/G	1	17	20	1	3480
YDR214W/AHA1	N	Cochaperone	16	P	2717	2239	3550	4401	5222
YPL240C/HSP82	N		14	P/G	134	76	2224	584	339
YBR155W/CNS1	Y	Cochaperone	13	P/G	nd	nd	4695	73	3188
YJR032W/CPR7	N	Cochaperone	13	P/G	nd	nd	3806	4235	3352
YKL117W/SBA1	N	Cochaperone	11	P	224	103	971	2097	124
YDR168W/CDC37	Y	Cochaperone		P/G	nd	nd	4732	3104	990
YLR216C/CPR6	N	Cochaperone	10	P	103	3674	4110	4243	2561
YOR057W/SGT1	Y	Cochaperone	10	P/G	nd	nd	2770	186	4909
YGR123C/PPT1	N	Cochaperone	6	P	1569	2583	4702	2687	447
YMR186W/HSC82	N		6	P/G	3637	3809	1992	5298	4652
YNL281W/HCH1	N	Cochaperone	6	P/G	3411	3601	5063	5376	4248
YCR060W/TAH1	N	Cochaperone	5	P	641	849	3951	4128	3069
YLR362W/STE11	N	Client	5	P	nd	nd	3189	2215	3519
YDR127W/ARO1	N		4	P	3214	3672	2579	4112	1920
YDR510W/SMT3	Y		4	P	nd	nd	3211	2279	3696
YGL073W/HSF1	Y	Client	4	P/G	nd	nd	5407	4881	5134
YHR027C/RPN1	Y		4	P/G	nd	nd	nd	nd	nd
YHR030C/SLT2	N	Client	4	P	nd	nd	nd	nd	nd
YNL064C/YDJ1	N	Cochaperone	4	P/G	nd	nd	5349	23	5
YCR077C/PAT1	N		3	P	nd	nd	nd	nd	nd
YDR283C/GCN2	N	Client	3	P	2122	2491	2144	5023	142
YDR328C/SKP1	Y		3	P/G	nd	nd	167	35	57
YER021W/RPN3	Y		3	G	nd	nd	5395	5370	5039
YFR052W/RPN12	Y		3	G	nd	nd	5326	5274	3015
YHR200W/RPN10	N		3	G	nd	3412	5132	738	4065
YIL075C/RPN2	Y		3	G	nd	3301	5347	5139	3868
YKR068C/BET3	Y		3	P	nd	nd	nd	nd	nd
YLR256W/HAP1	N		3	P	nd	nd	nd	nd	nd
YLR262C/YPT6	N		3	P/G	11	nd	76	338	4531
YML057W/CMP2	N		3	P	1527	3343	3464	2663	1346
YPL106C/SSE1	N	Cochaperone	3	P	nd	nd	1471	1798	5237

ORFs with three or more established interactions in BioGRID at the time of analysis are included in the table. If an ORF is essential or encodes a known Hsp90 cochaperone or folding client, that is indicated. The type(s) of Hsp90 interaction, physical (P) or genetic (G), is also specified for each ORF. For each screen and temperature condition, the numerical ranking of each ORF, out of all tested ORFs, with regard to sensitivity to Hsp90 inhibition is listed, with smaller numbers indicating a stronger growth defect. nd = not determined.

It was also difficult to identify unifying patterns of sensitivity for non-TPR containing Hsp90 cochaperones such as Aha1p, Sba1p, Cdc37p, and Hch1p. Indeed, yeast lacking one or both copies of *AHA1* or *HCH1* did not exhibit growth defects when Hsp90 was inhibited at any of the tested temperatures. The essential cochaperone Cdc37p, which in half-dosage did not result in sufficient growth inhibition to be included in any of the top 5% data sets, was most sensitive to Hsp90 inhibition at 37°C. On the other hand, homozygous deletion of the nonessential cochaperone Sba1p resulted in growth defects at both 30°C and 37°C, while in the heterozygous condition a single copy of *SBA1* was sufficient to permit robust growth in the presence of Hsp90 inhibition at 30°C, less well-tolerated at 15°C, and resulted in significant growth defects (i.e. inclusion in the top 5% data set) at 37°C.

We next examined whether any ranking patterns were common to established endogenous Hsp90 folding substrates Ste11p [48], Gcn2p [49], and Slt2p [38]. Unfortunately, the screens only generated usable data under all conditions for *GCN2* and only for half-dosage *STE11*; no ranked data for *SLT2* was obtained. Loss of one copy of *STE11* did not predispose yeast to significant growth defects upon Hsp90 inhibition at any of the three tested temperatures. *GCN2* loss led to hypersensitivity to Hsp90 inhibition only in the heterozygous condition at 37°C. Altogether, the limited number of known folding clients, and the lack of complete data for those established substrates in these screens, resulted in an inability to generate any testable screening patterns.

Overall, due to the lack of consistent or simply detected patterns for known cochaperones or folding clients, we were unable to successfully probe the data to reveal the candidates most likely to be novel Hsp90 cochaperones or substrates based solely on temperature and dosage-dependent rankings. This emphasizes the importance of subjecting these data sets to higher-order analyses exploring common molecular functions or cellular processes, as well as interaction network construction, to more successfully reveal robust insights into novel Hsp90 functions and substrates.

### Enriched gene ontology assignments amongst heterozygous data set ORFs

**Figure 2:**
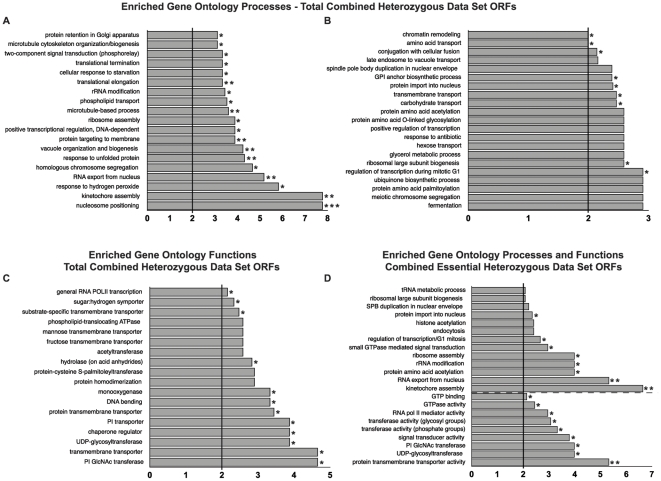
Significantly Enriched Gene Ontology Functions and Processes in Heterozygous Deletion Pool Data Sets. For each graph, only gene ontology classifications that were at least 2-fold enriched in the heterozygous data sets and for which at least three ORFs assigned to that term were tested are shown. Single asterisks (*) indicate p-value≤.05, double asterisks (**) indicate p-value≤0.005, and triple asterisks (***) indicate p-value≤0.0005. **A.** Most highly enriched Gene Ontology Process classifications in combined 15°C, 30°C, and 37°C heterozygous data sets. **B.** Additional enriched Gene Ontology Process classifications in combined 15°C, 30°C, and 37°C heterozygous data sets. **C.** Enriched Gene Ontology Function classifications in combined 15°C, 30°C, and 37°C heterozygous data sets. **D.** Enriched Gene Ontology Process classifications (above dashed line) and Gene Ontology Function classifications (below dashed line) for essential ORFs in combined 15°C, 30°C, and 37°C heterozygous data sets.

In order to establish whether specific cellular processes and functions are impacted by Hsp90 inhibition of heterozygous yeast deletion strains, the heterozygous data sets were analyzed with respect to Gene Ontology (GO) classifications. Based on criteria of at least a two-fold enrichment over expectations from a random selection of ORFs, as well as the stringent exclusion of categories for which fewer than three ORFs ascribed to that category were successfully tested in our screens, numerous enriched classifications emerge. Figure 2A depicts the most highly enriched (3.5 to 8-fold enrichment over background) and Figure 2B less highly enriched (2 to 3-fold enrichment over background) GO Process classifications, while Figure 2C delineates enriched GO Function assignments, amongst all combined heterozygous data set members. Of great interest, however, is focusing on GO assignments for essential ORFs in the heterozygous data sets. Enriched GO Process (Figure 2D, above dashed line) and GO Function (Figure 2D, below dashed line) assignments for only essential data set ORFs present some commonalities with results including all heterozygous data set ORFs, such as ribosomal large subunit biogenesis, ribosome assembly, rRNA modification, protein import into nucleus, and RNA export from nucleus. This analysis also reveals GO classifications uniquely enriched amongst essential data set members, including several GTP-binding and GTPase-related assignments, as well as RNA polymerase II mediator activity.

**Figure 3:**
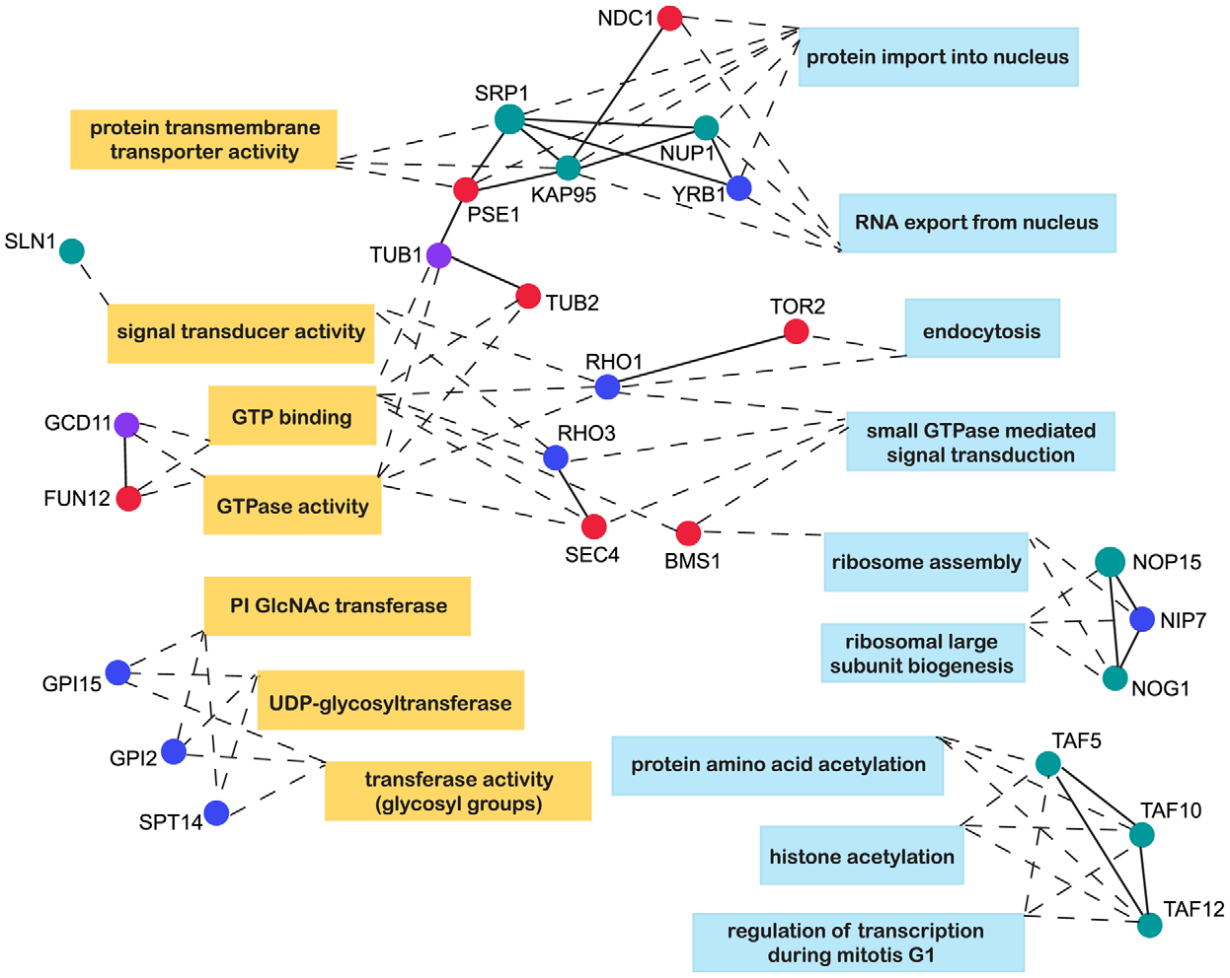
Essential Data Set ORFs Assigned to Multiple Significantly Enriched Gene Ontology Classifications. 15°C (teal), 30°C (blue), 37°C (red), and multiple temperature (purple) essential data set ORFs shown as nodes connected (by dashed lines) to significantly enriched Gene Ontology Functions (yellow rectangles) or Gene Ontology Processes (blue rectangles), to which they can be assigned. Solid lines indicate established physical or genetic connections between nodes. Node size correlates directly with number of times that ORF/protein has been previously identified as an Hsp90 interactor.

With the goal of providing further meaningful context for essential data set ORFs, we next focused on essential ORFs assigned to multiple enriched GO classifications. Essential data set members fulfilling this criterion are shown in Figure 3 connected with dashed lines to relevant GO Function (yellow boxes) and GO Process (blue boxes) designations. While some essential data set members are only affiliated with multiple GO Functions (i.e. GPI15, GPI2 and SPT14) or multiple GO Processes (i.e. TAF5, TAF10 and TAF12), numerous candidates bridge one or more GO molecular function or biological process categories. This clarifies, for example, that the enriched GO molecular function of protein transmembrane transporter activity is specifically referring to protein import into and RNA export out of the nucleus. Furthermore, connections are established between the function of GTP binding proteins and associated cellular processes such as endocytosis and ribosome assembly. Therefore, this analysis provides a more concrete look at GO assignments for essential data set ORFs, highlighting specific function/process connections for which a role for Hsp90 can be directly tested.

### Network analyses of heterozygous deletion pool data sets

**Figure 4:**
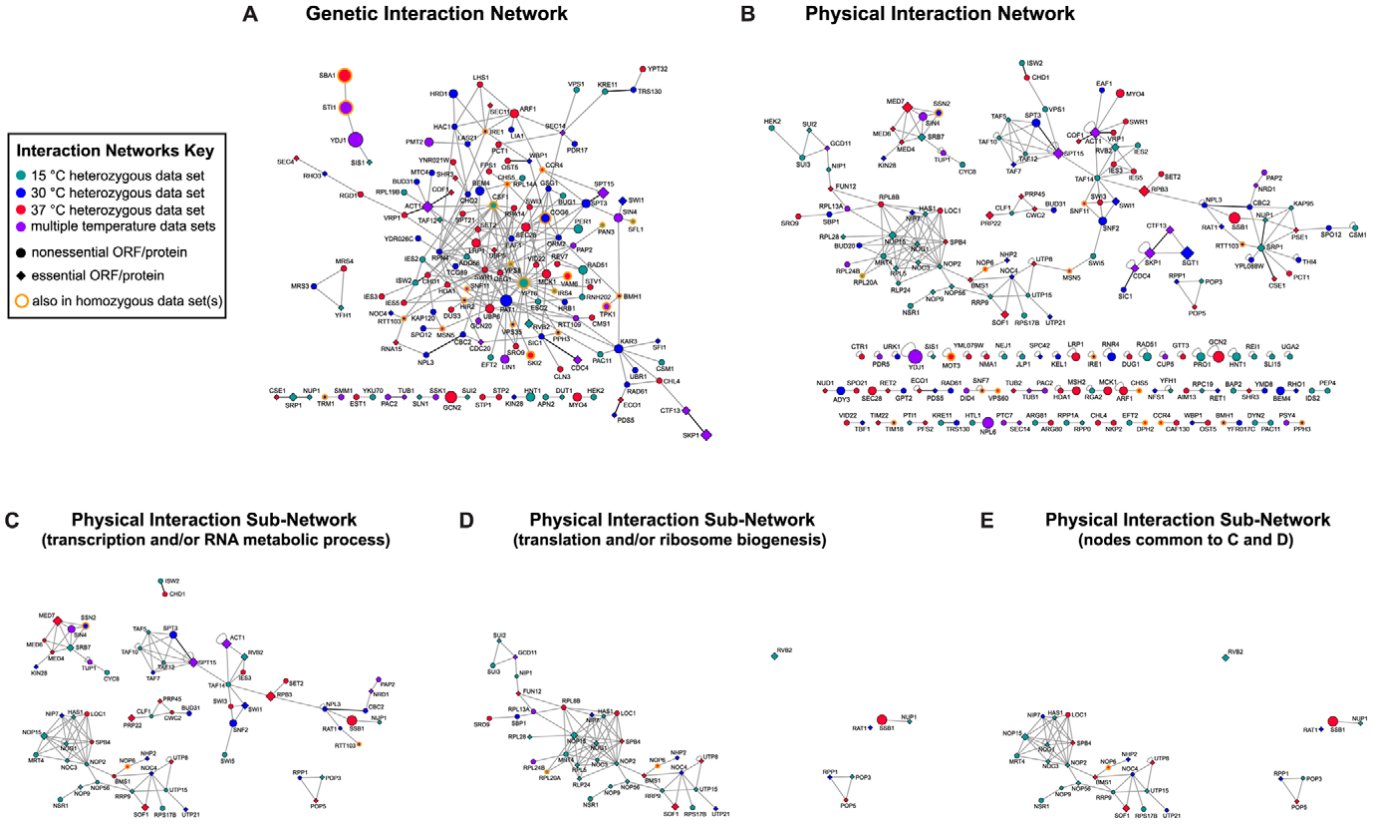
Integrated Network Analysis of Heterozygous Deletion Pool Data Sets. For all networks, node size directly correlates with confidence of Hsp90 interaction (number of times each ORF has been reported as an Hsp90 interactor). **A.** Genetic interaction network including all heterozygous deletion strain data set ORFs having curated genetic interactions amongst them. Bold edges indicate that there is also a curated physical interaction between those nodes. Less connected nodes are at the bottom of the figure. **B.** Physical interaction network including all heterozygous deletion strain data set ORFs having curated physical interactions amongst them and/or curated self-interactions. Bold edges indicate that there is also a curated genetic interaction between those nodes. Less connected and “loner” nodes (only self-interacting) are at the bottom of the figure. **C.** Physical interaction network nodes with GO assignments of transcription and/or RNA metabolic process. **D.** Physical interaction network nodes with GO assignments of translation and/or ribosome biogenesis. **E.** Physical interaction network nodes with GO assignments of transcription and/or RNA metabolic process and translation and/or ribosome biogenesis.

We next constructed networks in order to visualize how deletion strains affected by Hsp90 inhibition in the context of single gene dosage interact with one another. This process was initiated using curated physical and genetic interactions maintained in the BioGRID database [50]. Curated genetic interactions between heterozygous data set members are detailed in Figure 4A, while physical interactors in the data sets are shown in Figure 4B. The attributes of these networks allow for simple access to multiple kinds of information. For instance, the degree of confidence for each node as an Hsp90 interactor is indicated by its size. As an example, consider the well-established Hsp90 cochaperone Sba1p located in the upper left area of Figure 4A, which had eight independently confirmed BioGRID interactions at the time the parameters for these networks were established. Compare this to the node for *YPT32* (located in the upper right hand area of Figure 4A), which has no confirmed BioGRID interactions with *HSC82* or *HSP82*, with the exception of our previously published deletion pool screen data sets [40], which were excluded from inclusion in node size assignments for these networks. Thus, larger node size correlates directly with increased degree of Hsp90-interacting confidence. These networks also allow for simple determination of several other features, such as whether a given ORF is essential (diamond-shaped) or nonessential (circular), and whether an ORF was also identified as hypersensitive to Hsp90 inhibition in the context of identically performed homozygous deletion pool screens [40]. Of the two networks, the physical network is of significant interest in this report as it allows for prediction of proteins, perhaps essential proteins, which may directly interact Hsp90. All nodes in the physical interaction network genetically interact with Hsp90 as that was the basis of this screen, however, the observed growth defects upon Hsp90 interaction could also be due to the loss of a physical interaction with Hsp90 as, for the heterozygous deletion pools, one copy of each gene is still present. In contrast to the genetic network, the physical network is distinctly modular, with clearly defined ORF clusters that may represent specific cellular functions or processes. To investigate this further, the primary gene ontology categories affiliated with each node in the physical network were determined. This identified subnetworks of physical network nodes affiliated with GO assignments of transcription and/or RNA metabolic process (57.1% of physical network nodes; Figure 4C) or with GO assignments of translation and/or ribosome biogenesis (41% of physical network nodes). Several nodes are common to both physical subnetworks (Figure 4E); of these, all but five ORFs (*NUP1*, *RPS17*, *NOP6*, *NOP15*, and *SSB1*) are associated with RNA metabolic processes and all but one ORF (*SSB1*) are affiliated with ribosome biogenesis. Taking both essential and nonessential genes into account, as well as previously known and novel Hsp90 interactors, these network analyses of our data sets point to a significant role for Hsp90 in general transcription and translation, with an emphasis on RNA metabolic processes, perhaps as pertaining to ribosome biogenesis and assembly.

### Hsp90 interacts with BMS1 and is required for ribosome stability

**Figure 5:**
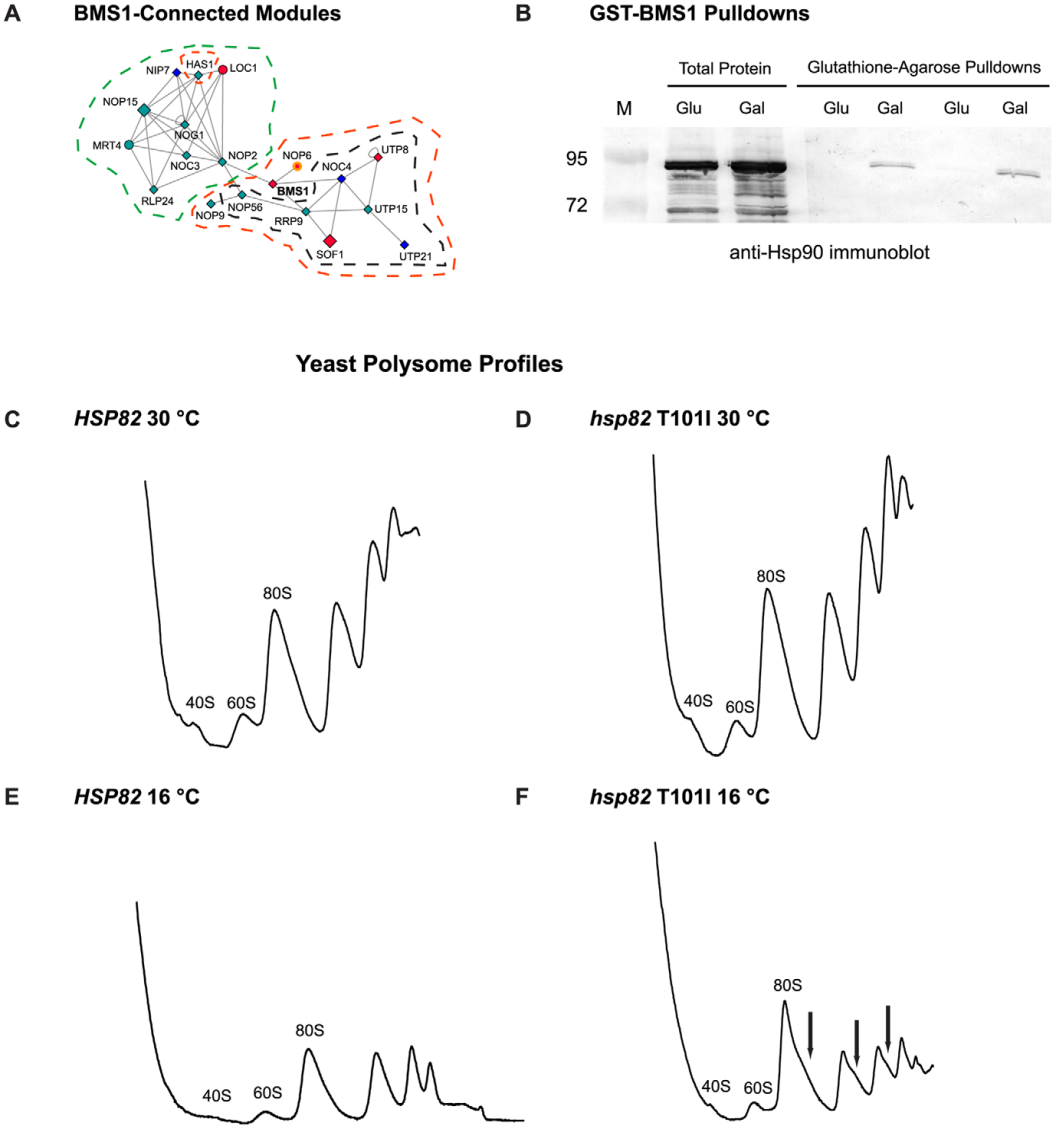
Hsp90 interacts with BMS1 and decreased Hsp90 function compromises 80S ribosome and polysome stability at 16°C. **A.** Physical interaction network modules connected by BMS1 (bold). Nodes are bounded according to their primary gene ontology macromolecular complex assignment(s): Green = Preribosome, large subunit precursor; Orange = 90*S* Preribosome; Black = Small subunit processome. **B.** Yeast expressing (Gal) or not expressing (Glu) GST-BMS1 were subjected to glutathione-agarose pulldowns and analyzed by SDS-PAGE and immunoblotting with anti-Hsp90 antisera. The left set of pulldowns were washed with 0.05% Triton-X 100, the right set of pulldowns were washed with 0.5% Triton-X 100. **C–F.** Yeast expressing only wild-type (**C** and **E**) or T101I (**D** and **F**) alleles of Hsp90 were grown at either 30°C (**C** and **D**) or 16°C (**E** and **F**) prior to ribosome isolation and profiling. Arrows indicate shoulders on the 80S ribosomal peak, as well as on subsequent polysome peaks (**F**).

In Figure 3, connections of essential data set ORFs affiliated with multiple primary GO assignments suggest essential cellular functions and processes that require Hsp90 activity. For example, there are several essential GTPases, among them BMS1, which is uniquely affiliated with the GO process of ribosome assembly [51], [52], [53]. Further, in the physical interaction network (Figures 4 B, C, D, and E), BMS1 appears as an essential bottleneck node linking a module consisting of macromolecular complex assignments of 90*S* preribosome (Figure 5A, orange-bounded nodes) or small-subunit processome (Figure 5A, black-bounded nodes) to another module assigned as preribosome, large subunit precursor (Figure 5A, green-bounded nodes). We therefore experimentally tested whether together these results engender a valid prediction of Hsp90 involvement in ribosome biogenesis or assembly.

First, co-immunoprecipitation experiments were performed to see whether Hsp90 physically interacts with Bms1p. Protein extracts from yeast harboring galactose-inducible GST-fused *BMS1*, grown in glucose or galactose, were subjected to glutathione-agarose affinity pulldowns. Immunoblot analysis of bound proteins revealed that Hsp90 co-purifies with GST-Bms1p, suggesting that Hsp90, either directly or indirectly, could be targeted to ribosomes along with Bms1p (Figure 5B).

Second, we asked whether conditions which compromise protein translation, in this case growth at 16°C, reveal a requirement for robust Hsp90 function with regard to ribosome stability. To this end, ribosome profiling experiments were conducted with yeast containing either wild-type or T101I mutant Hsp90 [54]. While at the optimal growth temperature of 30°C ribosome profiles from both wild-type and T101I mutant yeast yielded tight 40*S*, 60*S*, 80*S*, and polysome peaks (Figure 5C and D, respectively), growth at 16°C resulted in 80*S* and polysome instability in T101I yeast (Figure 5E, arrows).

Taken together, these results indicate that candidates, such as *BMS1*, that occur in data sets and are further suggested as important through GO and network analyses, can successfully instruct experimental efforts to validate roles and/or interactors of Hsp90.

### Identifying critical hubs of Hsp90 interaction: integrating essential data set ORFs and homozygous data set ORFs

**Figure 6:**
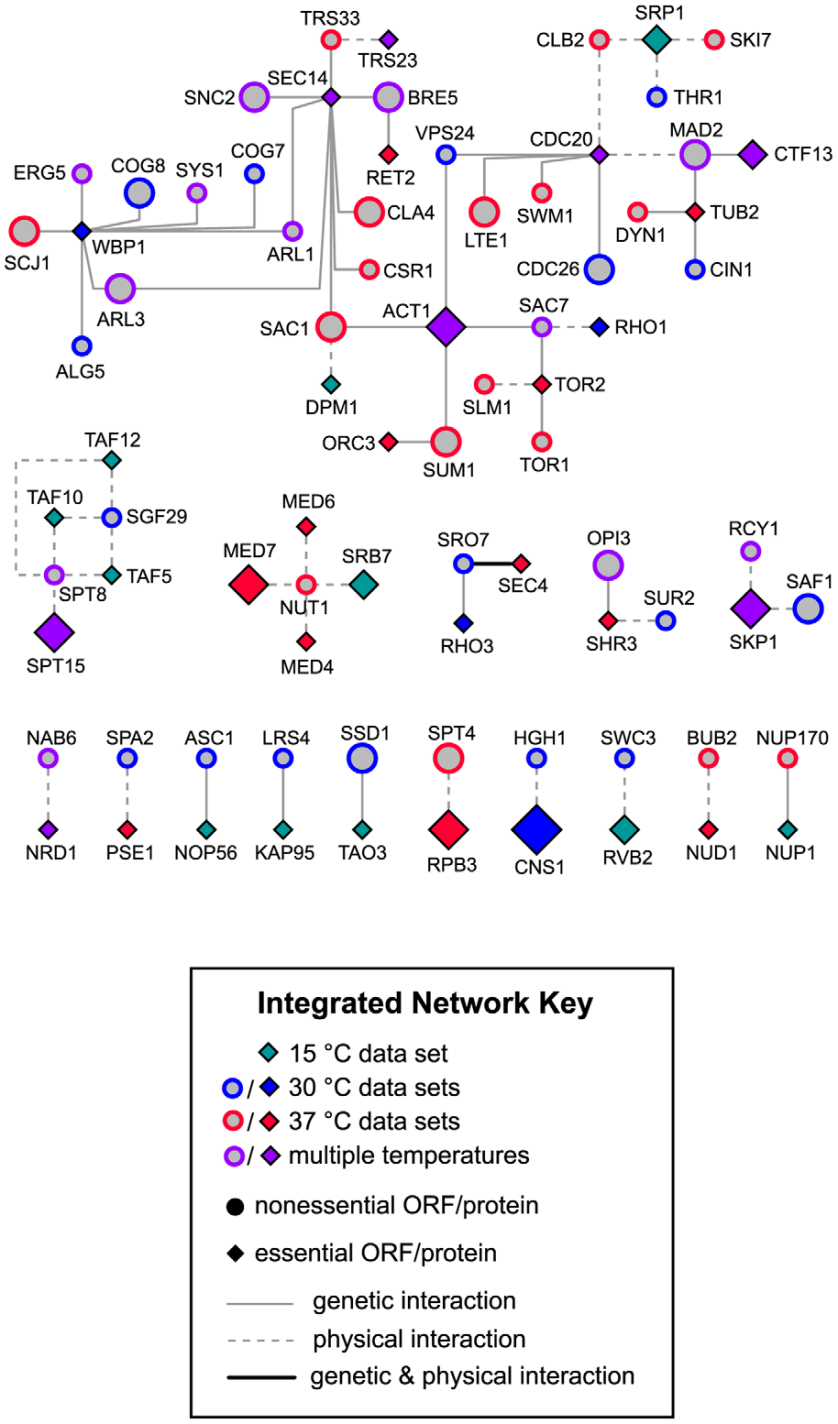
Integrated Network Connecting Essential ORFs and Homozygous Deletion Screen Data Set ORFs. This network depicts all essential heterozygous deletion strain data set ORFs with established connections to homozygous deletion strain data set ORFs. As described above, node size correlates with confidence of Hsp90 interaction.

Our final figure illustrates connections between essential ORFs identified in the heterozygous deletion pool screens and nonessential ORFS identified in identical screens conducted with homozygous deletion pools (Figure 6). This may establish essential targets of Hsp90 action or essential connections that are dramatically affected under conditions of Hsp90 inhibition. Of note, there are many instances in which an essential data set node serves as a hub of interaction, for example, the essential karyopherin-encoding gene *SRP1* connects to *CLB2*, *SKI7*, and *THR1*, three nonessential nodes from homozygous data sets. Similarly, the nodes for cytoskeletal proteins Act1p and Tub2p serve as hubs for numerous nodes. There are also instances of modules that alternately connect essential and nonessential nodes, as seen for the nodes for essential genes *TAF12*, *TAF10*, *TAF5*, and *SPT15*, which can be connected to one another via nonessential nodes *SGF29* and *SPT8*. There are also modules in which a nonessential gene identified in homozygous deletion pool screens, such as *NUT1*, serves as an interaction hub for essential proteins, in this case, as the central connector for mediator complex components Med7p, Med6p, Srb7p, and Med4p. Thus, genetic interactions gleaned from analyses of homozygous deletion pool data sets integrate well with and strengthen the validity of our identification of essential ORFs haploinsufficient to allow robust growth in the face of Hsp90 inhibition.

## Discussion

Herein we present the results from a meta-analysis of genome-wide chemical-genetic screens performed on the diploid yeast deletion collections in the absence and presence of the Hsp90 inhibitor macbecin II. Importantly, this robust analysis includes for the first time a screen conducted on heterozygous deletion strains at 15°C, in combination with previous heterozygous deletion pool screens conducted at 30°C and 37°C. This multi-temperature approach allows for explorations of condition-dependent cellular requirements for Hsp90. Of critical significance, it also permits the analysis of how both essential and nonessential half-dosage gene deletions contribute to compromised growth in the face of Hsp90 inhibition, revealing numerous essential genes and pathways that are affected.

When broadly comparing the data sets from the heterozygous deletion screens (Figure 1B) we found much less overlap between the different temperature data set ORFs than what was observed for homozygous deletion screens [40]. For example, while there was 29% overlap between 30°C and 37°C homozygous data sets, there is only 13.4% overlap amongst the 15°C, 30°C, and 37°C heterozygous data sets. This is perhaps indicative of more distinct temperature-sensitive requirements for Hsp90 under conditions of single copy gene deletion. A further difference between the broad analysis of homozygous and heterozygous deletion strain data sets is that numerous nonessential deletion strains were found to be hypersensitive to Hsp90 inhibition at 37°C in half-dosage, but not when both copies were deleted. This suggests the exciting possibility that these candidates represent labile Hsp90 clients that are dependent upon Hsp90 function under heat-stress conditions. The observed difference in Hsp90 requirement between homozygous and heterozygous states could be due to adaptations that have occurred in the complete absence of the protein versus growth defects that become apparent upon loss of folding maintenance and function of an existing protein. A well-established example of this in yeast is the case of SRP (Signal Recognition Particle), which is required for proper recognition and targeting of nascent secretory proteins to the ER membrane for cotranslational translocation. If SRP-encoding genes are deleted, yeast are still viable (albeit with translocation defects and slowed growth; [55]). Yeast instead harboring a temperature-sensitive allele of *sec65*, which encodes an essential protein subunit of SRP, are inviable at the non-permissive temperature [56], [57].

On the other hand, deletion strains that were hypersensitive to Hsp90 inhibition in the homozygous screens, but not when present in single copy, may indicate genetic or indirect, rather than direct physical Hsp90 interactors. For example, there is extensive evidence supporting a role for Hsp90 in intracellular vesicle transport [58], [59]. In mammalian cells, loss of Hsp90 recruitment to VAP-33 via the protein TPR1 disrupts intra-Golgi trafficking. The closest yeast homologue of VAP-33 is the ER membrane protein *SCS2*, which, like VAP-33, would not be expected to be a direct interactor of Hsp90. Yeast lacking both copies of *SCS2* are extremely hypersensitive to Hsp90 inhibition (i.e. in top 5% data sets at both 30°C and 37°C; [40]), however, in heterozygous screens the *SCS2::scs2Δ* strain is unaffected by Hsp90 inhibition at all three temperatures tested. This supports the supposition that genetic interactions or indirect/mediated physical interactions with Hsp90 are perhaps best detected by the homozygous screens, while direct physical interactors are better revealed by the heterozygous screens. There are, of course, obvious exceptions to this as Sti1p is known to directly bind Hsp90 and the lack of *STI1* in both homozygous and heterozygous contexts severely compromises growth when Hsp90 is inhibited. Thus, perhaps a modification to the above prediction is the exception of Hsp90 cochaperones as they both physically interact with Hsp90 and would be expected to exhibit genetic consequences as well since the Hsp90 chaperone cycle is highly dependent on appropriate interactions with its cochaperones. In any case, to gain further insight into these differences it will be of interest to compare the growth of relevant homozygous and heterozygous deletion strains as individual strains, outside of the context of the pools, in the presence of Hsp90-inhibiting drug to rule out non-specific effects. The hypotheses put forth above can also be directly tested by evaluating, for subsets of relevant homozygous and heterozygous deletion strains, if the affected proteins are indeed more likely to physically interact with Hsp90.

Regarding Hsp90 cochaperones, our finding that not all Hsp90 cochaperones, indeed not even those with a shared Hsp90 interacting motif (i.e. TPR domains), exhibit similar patterns of temperature and dosage-dependence (Table 3) points to the existence of combinatorial Hsp90-cochaperone interactions that could further dictate specificity and functionality of Hsp90. This is supported by recent observations that not only do asymmetric Hsp90-cochaperone complexes containing different TPR-cochaperones bound to each subunit of an Hsp90 dimer exist, but they in fact appear to be favored for forward progression of the Hsp90 chaperone cycle [60].

Of the three high-confidence Hsp90 cochaperones encoded by essential genes, the *CDC37::cdc37Δ* strain is most affected by Hsp90 inhibition at 37°C, while *CNS1::cns1Δ* and *SGT1::sgt1Δ* strains are strongly affected only at 30°C. While the latter two exhibit similar patterns and do both contain TPR domains, it has been shown that Sgt1p interacts with Hsp90 via a CS motif (CHORD-Sgt1; [47]). It is unclear whether any significance can be ascribed to these observed differences and similarities. Such an analysis is further complicated by the fact that not all cochaperones are expressed at similar levels, therefore, half-dosage likely means something completely different for each cochaperone under the different temperature conditions.

Predicting the most likely functional interactors or targets of Hsp90 with higher order analyses utilizing GO assignments and interaction networks was much more insightful. As already mentioned, one benefit of conducting these screens with the heterozygous deletion strains is the possibility of directly identifying physical interactors of Hsp90. The network analyses conducted using the heterozygous data sets readily support this notion. The physical interaction network (Figure 4B) is strikingly modular, indicating that if Hsp90 directly targets even one subunit of a protein complex, other subunits within that complex will also be affected by loss of Hsp90 function. This points to the existence of Hsp90 substrate “modules” in the cell and indicates a clear partitioning of known protein complexes into Hsp90-mediated or Hsp90-independent. Of note, this indication was previously suggested from generating a secretory pathway-specific physical interaction network from 30°C ad 37°C homozygous and heterozygous data sets, which was primarily populated with modular, cellular transport-related, protein complexes [40].

GO analyses of heterozygous data sets revealed several expected classifications, such as those related to the secretory pathway or cell cycle regulation, but also many intriguing new enrichments, such as nuclear transport, general transcription, ribosome assembly, and GTPase-related assignments. Importantly, these classifications were evident when limiting the analysis to essential ORFs only, suggesting that they underlie essential cellular requirements for Hsp90.

Regarding the connection between Hsp90 and nuclear transport, it is more likely the case that Hsp90 requires access to the nucleus for some of its activities rather than that Hsp90 is required for nuclear transport to occur. For example, Hsp90 translocates to the nucleus in association with its hormone receptor folding clients and Hsp90 cochaperone p23 assists the remodeling of associated transcriptional complexes [61], [62]. Hsp90 has also been shown to enter the nucleus in response to heat shock in mammalian cells [63], [64], [65] and in response to starvation in yeast [66]. As such, we would expect nuclear transport GO assignees identified in these screens to perhaps be assisting the nuclear transport of Hsp90. Indeed, strains heterozygous for the karyopherin genes *KAP95* and *SRP1*, recently found to assist the nuclear translocation of Hsp90 in yeast [66], are among those identified in our screens as hypersensitive to loss of Hsp90 function. Of interest, *KAP95* and *SRP1* both occur in the heterozygous 15°C data set, are highly ranked at 30°C (*KAP95* top 7.6%; *SRP1* top 16%), and less sensitive at 37°C (*KAP95* top 31%; *SRP1* top 40%). This suggests that Hsp90 does not require nuclear transport under hyperthermic stress (see also [66]), in contrast to what is observed for mammalian cells. Further, it suggests that Hsp90 may require access to the nucleus in more instances than just starvation, for instance, during hypothermic stress.

What might Hsp90 be required for in the nucleus? One likely possibility is a broader role for Hsp90 than previously thought in general transcription, most likely through assisting chromatin remodeling processes. This was first suggested by the above-mentioned paper by Freeman and Yamamoto (2002), further supported by the identification of chromatin remodeling complex components Tah1p and Pih1 as Hsp90-interacting proteins [39], and significantly bolstered in this report by the following: First, chromatin remodeling appears as a significantly enriched GO assignment in our analysis (Figure 2B). Second, our data sets engendered evident physical network modules for (1) mediator complex components, (2) various TAFs, and (3) numerous members of the SWI/SNF chromatin remodeling complex (Figure 4B). Of note, while neither *TAH1* and *PIH1* homozygous nor heterozygous deletion strains were sensitive enough to Hsp90 inhibition to be included in our data sets for analysis, the associated essential gene *RVB2* appears in the heterozygous 15°C data set. A role for Hsp90 in chromatin remodeling has been suggested to explain, at least in part, the dramatic phenotypic variation that can occur when the buffering capacity of Hsp90 is compromised [67]. Here we provide robust evidence for a connection between Hsp90 and the SWI/SNF chromatin remodeling complex, five members of which appear in our top 5% data sets (*SNF11*, *SNF12*, *SNF2*, *SWI1*, *SWI3*), as well as *SNF12* ranking in the top 8.3% of the 37°C heterozygous data sets and *SWP82* ranking somewhat lower, but exhibiting an identical pattern of sensitivity to *SNF11*. Whether Hsp90 and the SWI/SNF complex contribute independently to chromatin remodeling or act in conjunction remains to be determined. One could also envision that loss of appropriate transcriptional regulation or fidelity could similarly contribute to the expression of buffered phenotypes that is observed when Hsp90 function is compromised; the observed growth effects on strains heterozygous for general TAFs and mediator complex components in the presence of Hsp90 inhibitors suggest this as a possibility.

The chromatin remodeling components Tah1p and Pih1p, along with AAA-ATPases Rvb1p and Rvb2p, are also linked to snoRNP biogenesis [68], [69], and a role for Hsp90 in this process was recently confirmed [70]. We chose to specifically address a subset of this type of assembly in this report, namely ribosome assembly. Multiple ribosome assembly-related GO assignments were enriched in our analysis and, further, very clear functional modules related to ribosome assembly appear in our physical interaction network. Specifically, in the most minimal physical network (Figure 4E), the two main remaining modules (connected by essential ORF *BMS1*) are primarily composed of macro-molecular complex assignments of preribosome, large subunit precursor and 90*S* preribosome or small-subunit processome (highlighted in Figure 5A). Our experimental follow-up on these observations demonstrated that compromising Hsp90 function *in vivo* resulted in decreased polysome stability (Figure 5 C–F). A role for Hsp90 in maintaining ribosome stability in mammalian cells was also previously suggested [71], and mammalian ribosomal proteins were identified in proteomic screens for Hsp90 interactors [72], [73]. Altogether, we propose that Hsp90 may play a more significant role in ribosome biogenesis and stability than previously thought.

Lastly, we wish to consider the significant enrichment of GTPases identified in these chemical-genetic screens. Hsp90 has been implicated in Rab GTPase recycling in yeast and mammalian secretory pathways [58], and, more recently, shown to interact with Rab11a to regulate membrane recycling of alpha-synuclein [74]. Extensive connections between Hsp90 and the yeast secretory pathway were identified in our previous study, including six Rab family GTPases and five other transport-related GTPases [40]. The data and analyses presented here identify 24 GTP binding proteins in total, including six that are essential, that appear in at least one of our screen data sets. In addition to the previously mentioned transport-related GTPases, there are three GTPases involved in cell polarity, six linked to ribosome biogenesis or translation, and three that participate in other cellular processes. Altogether, these data point to a more important and pervasive connection between Hsp90 and cellular GTPases than previously realized and help to explain the far-reaching pleiotropic effects of inhibiting Hsp90 function in eukaryotes.

## Materials and Methods

### Growth and Treatment of Pooled Heterozygous Yeast Deletion Strains

Bar-coded heterozygous diploid yeast deletion strains in the BY4743 background [75] were grown in YPD (1% yeast extract, 2% peptone, 2% glucose) for ten generations at 15°C in the absence (no drug control; DMSO only) or presence of 200 µM macbecin II (in DMSO; courtesy of National Cancer Institute, Drug Synthesis and Chemistry Branch). Equivalent numbers of cells were harvested, snap-frozen in liquid nitrogen, and stored at −80°C until processing. Preparation of genomic DNA, PCR amplification of gene-specific molecular barcodes, hybridization to Tag3 arrays (Affymetrix, Santa Clara; generously provided by Ron Davis, Stanford University), and array visualization and analysis were all as previously described [40]. All growth, hybridization, and analyses were performed three times and the final data averaged together to generate average ratio values (Rvs) for each heterozygous deletion strain (Rv = average signal intensity in the presence of drug/average signal intensity in the absence of drug).

### Determination of Significantly Enriched GO Processes and Functions

Combined heterozygous deletion pool data sets (representing the top 5% most sensitive deletion strains from each of the three tested temperature conditions, 15°C, 30°C, and 37°C) were subjected to functional enrichment analyses using annotation data derived from FUNSPEC. The statistical significance of functional enrichments relative to ORFs tested in the heterozygous deletion screens was determined by Fisher's exact test. GO categories were designated as significantly enriched only if they met the following conditions: (1) sensitive ORFs were at least 2-fold enriched for the category relative to tested ORFs, and (2) at least three ORFs assigned to each GO category were successfully tested (i.e. yielded valid triplicate hybridization data) in the screens.

### Construction of Heterozygous Data Set Interaction Networks

We constructed physical and genetic interaction networks among the sensitive ORFs from our screens based on interaction data from BioGRID [50]. In order to produce high-confidence networks, we only included interaction edges with support from at least two separate experiments—sensitive ORFs with no confirmed interactions were excluded from the networks. Nodes are sized to reflect their total number of independent reports of interaction (physical and genetic) with Hsp90, not counting reports from our previous work [40]. Nodes are colored to reflect the screens in which their associated ORFs were sensitive: teal corresponds to sensitivity at 15°C, blue corresponds to sensitivity at 30°C, red corresponds to sensitivity at 37°C, and purple corresponds to sensitivity at multiple temperatures. Diamond-shaped nodes correspond to ORFs reported as essential (null phenotype) in the *Saccharomyces* Genome Database. Nodes with orange outlines correspond to ORFs that were sensitive in our previous homozygous deletion screens [40]. Black edges between nodes indicate that both genetic and physical interactions have been confirmed between the corresponding ORFs. In Figure 6, we illustrate physical and genetic interactions between essential ORFs from the three temperature heterozygous screens and nonessential ORFs from our previous homozygous screens. Homozygous sensitive nodes are indicated by a grey fill, with their temperature sensitivities indicated by border color (following the color scheme introduced above). Dashed edges correspond to confirmed physical interactions, and solid gray edges correspond to confirmed genetic interactions. These figures were generated in Cytoscape [76].

### GST Pulldown Experiments

BY4741 (MATa) yeast containing galactose-inducible GST-fused *BMS1* on a *URA3*-marked plasmid [77], [78] were grown to an OD_600_ of 1 in –URA dropout media (0.68% yeast nitrogen base lacking amino acids and 0.2% amino acid dropout mix lacking uracil) containing either 2% glucose or 2% galactose. 20 ODs of cells were harvested, washed once with cold sterile distilled water, and resuspended in 500 µL of GST Buffer (25 mM Tris pH 8.0, 150 mM NaCl, 5 mM MgCl_2_, 1 mM EDTA, 2.5% glycerol, 0.05% Triton X-100) supplemented with fresh 1 mM DTT and protease inhibitors. 100 µL of acid-washed glass beads (G8772; Sigma) were added and cells were ruptured by vortexing for 10 minutes at 4°C. Cell lysates were clarified by spinning at 13,000 rpm in a microcentrifuge at 4°C for 10 minutes. Clarified protein extracts (supernatants) were quantified using Bradford reagent (Biorad, Hercules, CA) and an A_595_ filter in a plate reader (Infinite F200; Tecan, Switzerland). 4 mg of total protein were incubated with rotation at 4°C in a total volume of 500 µL with 40 µL of a 50∶50 glutathione-agarose slurry (Gold Biotechnology, St. Louis, MO). Glutathione-agarose beads were washed four times with 500 µL of GST Buffer containing either 0.05% or 0.5% Triton X-100. Washed beads were resuspended in 40 µL of 2X SDS sample buffer, heated at 85°C for 10 minutes, and loaded onto 12% SDS-PAGE gels (Hoefer, Holliston, MA). Gels were transferred onto nitrocellulose (Biorad) using a semi-dry transfer apparatus (BioRad) for 45 minutes at 15 V. The blocked nitrocellulose filter was probed with polyclonal Hsp90 antisera (kind gift of Avrom Caplan, CCNY) and HRP-conjugated secondary antibody (goat anti-rabbit HRP; Biorad), and developed colorimetrically (Opti 4CN Substrate Kit, Biorad).

### Ribosome Profiling

200 mL of yeast in exponential growth phase were treated with 100 µg/mL of cycloheximide, harvested, washed with cold lysis buffer (20 mM Hepes pH 7.5, 50 mM KCl, 10 mM MgCl_2_, 1% Triton X-100), resuspended in 1 mL of buffer A (20 mM HEPES pH 7.5, 50 mM KCl, 10 mM MgCl_2_, 1% Triton X-100, 1 mM DTT, and protease inhibitors) and finally frozen as drops in liquid nitrogen. The cells were then ground using a MM301 grinder (Retsch®, Germany). 0.5 mL of buffer A was then added to the powder and the lysates were clarified by centrifugation at 14,000 g for 10 minutes at 4°C. 20 OD of lysate was applied on a 12 mL 7%–47% sucrose gradient in buffer A and centrifuged in a SW41 rotor for 150 minutes at 39,000 rpm at 4°C. Fractions were collected using a UA-6 detector (Teledyne Isco, Lincoln, NE).

## Supporting Information

Table S1 **Determination of statistical significance of various sensitivity cutoffs for data sets.** The number of total ORFs analyzed for each experimental condition was compared to the number of those ORFs annotated in BioGRID as known Hsp90 (*HSP82* and/or *HSC82*) interactors to determine the number of ORFs in common to both (overlap). The fold-enrichment and statistical significance of those ORFs occurring in data sets made from various cutoffs (top 5%, 7.5%, 10%, or 15% most sensitive) was then determined.
(XLSX)

Table S2 **15°C heterozygous deletion pool data set.** ORFs comprising the top 5% most sensitive heterozygous deletion strains (273 of 5421 tested ORFs) listed alphabetically by ORF name.
(XLSX)

Table S3 **Total ranked data from heterozygous deletion pool 15°C screen.** All 5421 ORFs tested are listed by average ratio value ranking from most sensitive (most growth compromised when Hsp90 is inhibited) to least sensitive.
(XLSX)

Table S4 **Essential heterozygous data set ORFs.** Heterozygous deletion strains with single copies of essential genes occurring in data sets of any of the three temperature screens (total of 155 ORFs) are shown. Grey coloration indicates the 19 essential ORFs previously annotated in BioGRID as known Hsp90 (*HSP82* and/or *HSC82*) interactors. Green coloration indicates the 136 ORFs representing novel essential Hsp90 interactors.
(XLSX)
